# Differential diagnosis of reactive arthritis in a patient with intravesical BCG immunotherapy

**DOI:** 10.1186/1471-2334-14-S7-P25

**Published:** 2014-10-15

**Authors:** Aida Roxana Aguridă, Cristina Maria Sîrbu, Alina Cristina Neguț, Maria Magdalena Moțoi, Luminița Mariana Bradu, Ramona Ștefania Popescu, Andrei Nițulescu, Anca Streinu-Cercel, Adrian Streinu-Cercel

**Affiliations:** 1 National Institute for Infectious Diseases "Prof. Dr. Matei Balş", Bucharest, Romania; 2 Carol Davila University of Medicine and Pharmacy, Bucharest, Romania

## Background

Intravesical administration of bacillus Calmette-Guerin, a live attenuated strain of *Mycobacterium bovis*, is an adjunctive therapy for superficial bladder cancer. While generally well tolerated, both local and systemic complications may appear. The dose of BCG in instilations is 100 fold higher than a BCG vaccine.

## Case report

We report the case of a 50 year-old female patient known with bladder tumor operated in January 2014 followed by 4 weeks of intravesical BCG immunotherapy, one administration per week. After each course the patient accused low grade fever, nausea, pollakiuria and hematuria with limited duration. In March 2014, after a cystoscopy and a bladder resection, she underwent another 5 weeks of BCG instillations. After the fourth course the patient presented to our clinic for fever with chills, pollakiuria, hematuria, conjunctivitis, myalgia and disabling migratory arthritis of the left ankle and right knee.

Clinical exam at admission: high fever, left ankle and right knee arthritis, impaired mobility in the left temporomandibular joint. Following admission the patient developed left metacarpophalangeal and proximal interphalangeal arthritis of the index and medius. She had leukocytosis with neutrophilia, reactive thrombocytosis and high biologic inflammatory syndrome. Urinalysis showed frequent leukocytes, no albuminuria and negative cultures.

The patient underwent arthrocentesis for synovial fluid sampling; the smear showed absence of bacteria, 40% polymorphonuclear cells, 15% small lymphocytes, 20% medium lymphocytes, 25% large lymphocytes and the Ziehl-Neelsen smear was negative. The culture for *Mycobacterium* spp. was negative.

Suspecting a BCG arthritis and cystitis we started empiric antituberculous (antiTB) and glucocorticoid therapy. A week after admission *Serratia marcescens* was identified in one blood culture out of three collected, so we added ertapenem for 14 days.

Rheumatological examination raised suspicion of an autoimmune illness, but all specific blood tests were negative. Despite that, the rheumatologist added sulfasalazine, considering that even BCG arthritis can associate an autoimmune disorder. After 2 months of antiTB, glucocorticoid and sulfasalazine treatment the evolution was favorable, with remission of arthritis and fever.

## Conclusion

In our case, the cause of arthritis could be the BCG instillations, an autoimmune illness or the infection with *Serratia marcescens*. While the long-term progression of symptoms despite antiTB treatment is a strong argument in favor of an autoimmune cause (negative specific tests being a counterargument), we cannot exclude the immune disorder caused by BCG immunotherapy.

